# Bactericidal Activity of Selenium Nanoparticles Against a Multidrug-Resistant Pathogen: Mechanistic Hypothesis from Exploratory Proteomics

**DOI:** 10.3390/microorganisms14010089

**Published:** 2025-12-31

**Authors:** Nora Elfeky, Jing-Ru Chen, Meng-Xiao Zhu, Jing-Dian Wang, Aya Rizk, Mohammed T. Shaaban, Guoping Zhu

**Affiliations:** 1 Anhui Provincial Key Laboratory of Molecular Enzymology and Mechanism of Major Metabolic Diseases, College of Life Sciences, Anhui Normal University, Wuhu 241002, China; jingruchen@ahnu.edu.cn (J.-R.C.); 2221011723@ahnu.edu.cn (M.-X.Z.); wjd920@ahnu.edu.cn (J.-D.W.); 2 Botany Department, Faculty of Science, Menoufia University, Shebin El-koom 32511, Egypt; aya.mohamed1701042@science.menoufia.edu.eg (A.R.); mhdtawfiek@gmail.com (M.T.S.)

**Keywords:** selenium nanoparticles, MDR, antimicrobial activity, proteomics analysis, glutathione system

## Abstract

The antimicrobial resistance crisis necessitates novel therapeutics. Selenium nanoparticles (SeNPs) offer promise, but their precise bactericidal mechanism remains poorly defined. This study aimed to define the antibacterial action of SeNPs synthesized via a green method with ascorbic acid and sodium citrate. The resulting SeNPs were monodisperse (17.8 ± 0.66 nm), crystalline, and highly stable (zeta potential: −69.9 ± 4.3 mV), exhibiting potent bactericidal activity against multidrug-resistant *E. coli*. To generate a mechanistic hypothesis, we integrated phenotypic analyses with a preliminary, single-replicate proteomic profiling. Recognizing this as an exploratory step, we focused our analysis on proteins with the most substantial changes. This revealed a coherent pattern of a targeted dual assault on core cellular processes. The data indicate that SeNPs simultaneously induce oxidative stress while severely depleting key components of the primary antioxidant glutathione system; key detoxification enzymes—glutathione S-transferase and glutaredoxin 2—were depleted 18- to 19-fold, while the stress protein HchA was reduced by over 63-fold. Concurrently, the patterns point toward a crippling of central energy metabolism; iron–sulfur enzymes in the TCA cycle, including aconitate hydratase (8.1-fold decrease) and succinate dehydrogenase (13.9-fold decrease), were severely suppressed, and oxidative phosphorylation was impaired (e.g., 4.7-fold decrease in NADH dehydrogenase subunit B). We propose that this coordinated disruption creates a lethal feedback loop leading to metabolic paralysis. Consequently, this work provides a detailed and testable mechanistic hypothesis for SeNPs action, positioning them as a candidate for a potent, multi-targeted antimicrobial strategy against drug-resistant pathogens.

## 1. Introduction

Antimicrobial resistance (AMR) denotes a condition in which infectious microbes—bacteria, viruses, fungi, and parasites—develop diminished susceptibility to antimicrobial agents. The consequent drug resistance leads to the failure of standard treatment protocols, rendering infections increasingly recalcitrant to therapy [1,2]. This treatment failure amplifies the potential for widespread transmission of disease, severe illness, and patient mortality [1,3].

The genesis of AMR is rooted in the natural genetic adaptation of microorganisms. However, the primary driver for its rapid emergence and dissemination is human activity [1]. The selective pressure exerted by the extensive and often inappropriate application of antimicrobials in clinical, veterinary, and agricultural contexts is the most significant accelerating factor [1]. In this context, inorganic nanoparticles have emerged as promising alternatives due to their ability to target bacteria through physical and biochemical mechanisms that are distinct from traditional antibiotics, potentially overcoming existing resistance pathways [4,5]. Among these, selenium nanoparticles (SeNPs) have attracted significant interest due to their high biocompatibility and low toxicity compared to ionic selenium and other metal-based nanoparticles, alongside their demonstrated broad-spectrum bioactivities [6]. Selenium nanoparticles (SeNPs) offer significant advantages over other forms of selenium, making them highly attractive for biomedical applications. Compared to inorganic selenium salts like selenite and selenate, SeNPs exhibit a substantially lower toxicity profile, expanding the safe therapeutic window of this essential trace element [7]. Their nanoscale size contributes to greater bioavailability and a higher degree of biological activity, enhancing their absorption and efficacy [8]. These nanoparticles are notable for their biocompatibility and versatile functionality, as their surface can be easily modified or conjugated with various biological molecules, such as proteins or polysaccharides, to improve stability, targeting, and specific therapeutic effects [9,10]. Selenium nanoparticles (SeNPs) exhibit a remarkable dual functionality as both anticancer and antioxidant agents, with the specific outcome largely determined by the biological context and cellular redox environment. This dual behavior is central to their therapeutic appeal [11,12,13]. A particularly promising application of SeNPs is their potent and multifaceted antimicrobial activity. Research has demonstrated that SeNPs have a broad spectrum of action against both Gram-positive and Gram-negative bacteria, as well as fungi like *Candida albicans* [8,14]. Their efficacy is highly dependent on physicochemical properties, with studies showing that controlling particle size is critical for maximizing antibacterial effects; for instance, SeNPs around 81 nm in diameter were found to be optimal for inhibiting methicillin-resistant *Staphylococcus aureus* (MRSA) [15].

However, the antimicrobial efficacy of SeNPs is highly dependent on their physicochemical characteristics, such as size, crystallinity, and surface chemistry, which are, in turn, dictated by the synthesis method [14]. Many conventional chemical syntheses involve harsh reducing agents and stabilizers, raising concerns about residual toxicity and environmental impact. Therefore, developing a green or environmentally benign synthesis that utilizes non-toxic reagents to produce well-defined SeNPs is a critical step toward their safe biomedical application. To this end, this study employs a synthesis strategy using ascorbic acid (Vitamin C) as a reducing agent and sodium citrate as a stabilizer. Ascorbic acid is a biocompatible and potent reductant, ideal for generating SeNPs without introducing toxic by-products [16,17]. Similarly, citric acid and its salts act as an effective capping agent to control particle growth and prevent aggregation, ensuring colloidal stability and enhancing biocompatibility [18].

Despite the growing body of literature on the antibacterial properties of SeNPs [19], more knowledge is still needed to understand their precise mechanism of action at the molecular level on the bacterial cells. Many studies describe phenotypic outcomes, such as growth inhibition and membrane damage [19], but the subsequent intracellular events and the global metabolic response of the bacterial cell remain poorly characterized. This lack of mechanistic depth hinders the rational design and application of SeNPs as effective antimicrobials.

To address this gap, we employed a preliminary proteomic analysis to formulate a specific, testable mechanistic hypothesis for the cytotoxic effects of our ascorbic acid/citrate-synthesized SeNPs on MDR bacteria. Quantitative proteomics provides a powerful, unbiased tool to map the entire landscape of protein expression changes in response to stress, revealing the specific cellular pathways and processes that are targeted [20]. This study aimed to fabricate selenium nanoparticles (SeNPs) using a straightforward, green synthesis method involving the reduction of sodium selenite with ascorbic acid and sodium citrate. The primary objectives were to evaluate the antibacterial efficacy of these SeNPs against multidrug-resistant bacteria and to employ an initial, exploratory proteomic approach to identify coherent molecular patterns. The resulting data were used to generate a testable hypothesis regarding the antimicrobial mechanism, providing a foundational framework for future development of SeNP-based therapies against MDR pathogens.

## 2. Materials and Methods

### 2.1. Microorganisms

The test pathogens *Pseudomonas aeruginosa* (ATCC27853), *Staphylococcus aureus* (ATCC12600), *Escherichia coli* (ATCC13706), *Klebsiella pneumoniae* (ATCC43816), and *Escherichia coli* (ATCC25922) were from the American Type Culture Collection (ATCC, Rockville, MD, USA). And MDR urinary tract infection pathogens, *Enterococcus* sp. N2023 and *Escherichia coli* Mar002 were provided by the Microbiology laboratory of Menoufia University, Egypt. To prepare overnight broths of each microorganism, one colony was suspended in Luria–Bertani broth (LB, Thermo Fisher, Waltham, MA, USA), incubated at 37 °C with agitation at 200 rpm.

### 2.2. Molecular Identification of MDR UTI Pathogens

DNA was amplified via polymerase chain reaction (PCR) using the universal bacterial primers 27F (AGRGTTYGATYMTGGCTCAG) and 1492R (RGYTACCTTGTTACGACTT), targeting the 16S rRNA gene. The PCR was performed using a TransStart Fastpfu DNA Polymerase system (TransGen Biotech, Beijing, China) in a 50 μL reaction volume, containing 10 μL of 5× FastPfu Buffer, 2 μL of 2.5 mM dNTPs, 1 μL of each forward and reverse primer (5 μM), 0.5 μL of FastPfu Polymerase, and approximately 10 ng of template DNA, with the remaining volume made up with ddH_2_O. Amplification was carried out on an ABI GeneAmp^®^ 9700 PCR instrument with the following thermal cycling parameters for bacteria: an initial denaturation at 95 °C for 5 min, followed by 27 cycles of denaturation at 95 °C for 30 s, annealing at 55 °C for 30 s, and extension at 72 °C for 45 s, with a final extension at 72 °C for 10 min before holding at 10 °C. The resulting PCR products were purified and sequenced using the 3730XL DNA Analyzer. The obtained sequences were then compared against reference databases using the NCBI BLAST algorithm (https://blast.ncbi.nlm.nih.gov/Blast.cgi?PROGRAM=blastn&PAGE_TYPE=BlastSearch&LINK_LOC=blasthome, access 28 November 2025), where key parameters such as Max Score, Total Score, Query Coverage, and E-value were interpreted to assign taxonomic identity, with high scores and coverage and a low E-value indicating a reliable match. Phylogenetic analysis was conducted using MEGA 12 [21] software with the Neighbor-Joining method for tree construction [22]. The evolutionary distances were computed using the p-distance method. The complete deletion option was applied to eliminate positions containing gaps and missing data. Phylogenetic trees were visualized using the ITOL web server (https://itol.embl.de, access 28 November 2025).

### 2.3. Selenium Nanoparticle (SeNPs) Synthesis

Selenium nanoparticles (SeNPs) were fabricated using ascorbic acid (AA) as reducing agent and 1 mM sodium citrate (pH 7.5) as stabilizer. 10 mL of sodium selenite (Na_2_SeO_3_) (1 mM) was added to the reaction mixture, which consisted of 100 mL of 1 mM sodium citrate with pH adjusted to 7.5. Then, 30 mL of ascorbic acid (4 mM) was added dropwise to the reaction mixture with continuous stirring at 70 rpm. The color changed gradually to dark red. The flask was covered by foil and stirred for three hours, then incubated for 24 h at 30 °C and 200 rpm for complete reduction of Na_2_SeO_3_. SeNP formation was monitored visually by the color change to red and confirmed by UV-Vis spectroscopy. The nanoparticles were harvested by centrifugation at 12,000 rpm for 20 min. The pellets were washed twice with double-distilled water to remove impurities. The purified selenium nanoparticles were then lyophilized and stored in airtight, dark containers to prevent oxidation and degradation until further characterization.

### 2.4. Characterization of Nano Selenium Particles

- Transmission electron microscope

A drop of the SeNPs suspension was placed on the carbon-coated copper grids (CCG) and dried by allowing water to evaporate at room temperature. Electron micrographs were obtained using JEOL JEM-1010 transmission electron microscope (Tokyo, Japan) at 80 kV at the Regional Center for Mycology and Biotechnology (RCMB), Al-Azhar University [23].

b. Zeta potential Estimation

A stock concentration of 1 mg/mL of SeNPs was prepared. This stock solution was subsequently diluted 100-fold to prepare it for analysis. To ensure dispersion and disrupt any aggregates, the diluted samples were subjected to sonication for a duration of 5 min. The zeta potential was then quantitatively assessed using Dynamic Light Scattering (DLS) performed on a Zetasizer Nano ZN instrument (Malvern Panalytical Ltd., Malvern, UK). All measurements were conducted at a fixed scattering angle of 173° and a temperature maintained at 25 °C, with each sample analyzed in triplicate to ensure statistical reliability.

c. X-Ray Diffraction (XRD) Analysis

The crystalline structure and phase identification of the selenium nanoparticles were performed on a Bruker D2 Phaser 2nd Gen diffractometer (Billerica, MA, USA) equipped with a Cu X-ray tube anode (operating at 30 kV and 10 mA) and a monochromator. The Cu Kα radiation (wavelengths: α_1_ = 1.54060 Å, α_2_ = 1.54439 Å) was used. The powder sample was loaded onto a standard sample holder and spun at 15 rpm. Data were collected in a Bragg–Brentano geometry over a 2θ range of 10° to 70° with a step size of 0.04° and a dwell time of 132 s per step. Divergence and receiving slits were set to 1.00 and 2.5 mm, respectively. The analysis was conducted at an ambient temperature of 23.5 °C.

### 2.5. Antimicrobial Activity of Nano Selenium Particles

The antimicrobial efficacy of the synthesized selenium nanoparticles (SeNPs) was assessed against a panel of human pathogens using the disk diffusion method according to CLSI guidelines [24]. Bacterial lawns were prepared by streaking test organisms (5 × 10^5^ cfu/mL) onto Mueller–Hinton agar plates. Aliquots (10 µL) of the SeNPs suspension (15 µg) were applied to sterile 5 mm filter paper discs. Following drying, the discs were aseptically transferred onto the surface of the inoculated agar. For the negative control, discs were impregnated with 10 µL of a 0.1 mM sodium citrate solution at pH 7.5. All plates were subsequently incubated at 37 °C for 24 h [24]. Post-incubation, the diameter of the inhibition zones was measured. The assay was performed in triplicate for each condition, and data are expressed as the mean values with their corresponding standard deviations. Kanamycin was used as a positive control.

### 2.6. Assays for MIC and MBC Determination of SeNPs Against Different Pathogenic Microorganisms

The MIC of the selenium nanoparticles suspension was determined by micro-broth dilution method using Muller–Hinton broth. Briefly, overnight cultures grown on Mueller–Hinton agar were adjusted to around 5 × 10^−5^ colony-forming units (CFU) per milliliter of Mueller–Hinton broth. In a 96-well microtiter plate, 50 μL of this bacterial solution was mixed with 50 μL of the twofold-concentrated SeNPs colloid (600 µg/mL to 0.586 µg/mL). The plates were incubated at 37 °C for 20 ± 2 h [24]. The MIC values were considered as the lowest concentration of the SeNPs, which showed no turbidity. MBC was determined by subculturing 0.01 mL aliquot from macroscopically clear wells to sterile nutrient agar for 24 h at 37 °C. The MBC was defined as the lowest concentration of the SeNPs at which no bacterial colonies on MHA were observed. MBC/MIC ratio was calculated to determine bactericidal or bacteriostatic properties of SeNPs against bacterial pathogens [25]. Kanamycin was used as a positive control.

### 2.7. Preparation and Examination of Bacteria Using Transmission Electron Microscope

*E. coli* Mar002 strain was utilized as a model pathogen to assess the cytotoxic effects of SeNPs. The bacterial samples treated with 10 µg/mL SeNPs were processed for transmission electron microscopy (TEM) imaging, and their morphological changes were compared to untreated control cells. To prepare the samples, bacterial cells were harvested by centrifugation at 4000 rpm for 10 min from 24 h old cultures grown in nutrient broth, followed by washing with distilled water. The cells were then fixed in 3% glutaraldehyde, rinsed in phosphate buffer, and post-fixed in a potassium permanganate solution for 5 min at room temperature. Dehydration was performed using an ethanol series (10% to 90%) for 15 min at each concentration, followed by a 30 min treatment with absolute ethanol. The samples were then infiltrated with epoxy resin through a graded series of acetone and resin solutions. Ultrathin sections were collected onto copper grids and double-stained with uranyl acetate and lead citrate. The sections were examined under a transmission electron microscope (JEOL JEM-1010, Tokyo, Japan) at 80 kV at the Regional Center for Mycology and Biotechnology (RCMB), Al-Azhar University [26,27].

### 2.8. Proteomics Analysis

*E. coli* Mar002 samples, either control or treated with 10 µg/mL SeNPs, were used for proteomics analysis to understand the cytotoxicity of SeNPs on protein expression. Protein extraction was performed by adding 200 µL of 8 M urea buffer (500 mM Tris, pH 8.5) to the bacterial pellet samples, followed by homogenization using an ultrasonic homogenizer. The homogenate was then vigorously shaken and centrifuged at 10,000 RPM for 30 min at 4 °C [28]. The resulting protein supernatant was quantified using the Bradford assay on a deNovix DS-11 FX Series instrument [29] (Wilmington, DE, USA). For digestion, a total of 30 µg of protein from each sample was reduced with 2 µL of 200 mM dithiothreitol (DTT) for 45 min at room temperature and subsequently alkylated with 2 µL of 1 M iodoacetamide (IAA) for 45 min in the dark [28,30]. The volume was adjusted with 102 µL of 100 mM Tris (pH 8.5) before tryptic digestion was initiated by adding 6 µL of trypsin (1 µg porcine enzyme) and incubating overnight at 37 °C with shaking at 900 rpm. Digestion was halted by acidifying the samples to pH 2–3 with 6 µL of 100% formic acid [28,30].

Peptides were purified and desalted using MonoSpin Reversed-Phase Columns (Stage Tip). The columns were activated with 50 µL of methanol, initialized with 50 µL of solution B (0.2% formic acid, 80% acetonitrile), and re-equilibrated twice with 50 µL of solution A (0.2% formic acid). The acidified digest was loaded onto the column, washed twice with 50 µL of solution A, and peptides were eluted with three aliquots of 50 µL of solution B [29,30]. The eluate was concentrated using a speed-vac and reconstituted in 20 µL of solution A. The peptide concentration was determined using a bicinchoninic acid (BCA) assay on the deNovix DS-11 FX instrument [31]. For liquid chromatography–mass spectrometry (LC-MS/MS) analysis, 1 µg of peptides (in 10 µL) was injected into an Eksigent nanoLC 400 system coupled to a Sciex TripleTOF^TM^ 5600+ mass spectrometer (Framingham, MA, USA). Peptides were trapped on a CHROMXP C18-CL cartridge (5 µm, 10 × 0.5 mm) and separated on a ChromXP C18-CL column (3 µm, 120 Å, 150 × 0.3 mm) using a 57 min gradient from 3% to 80% mobile phase B (0.1% formic acid in acetonitrile) at a flow rate of 5 µL/min [28]. Data were acquired in positive ion mode with a TOF mass range of 400–1250 *m*/*z*. Information-dependent acquisition (IDA) consisted of a high-resolution TOF-MS survey scan followed by product ion scans of the top 40 ions (170–1500 *m*/*z*) with a cycle time of 1.5 s [28]. Raw data files were processed using Protein Pilot software (version 5.0.1.0) with the Paragon algorithm. Database searching was performed against the UniProt Escherichia coli database (4612 entries) with the following parameters: cysteine alkylation with iodoacetamide, tryptic digestion, a thorough search effort, biological modifications ID focus, and false discovery rate (FDR) analysis enabled with bias correction [28]. The resulting proteome content of each sample was compared to that of others for differential protein expression by means of fold change calculations. Significantly upregulated proteins (not less than 2-fold changes in treated sample) together with treatment-unique proteins were used for gene enrichment analysis using clusterProfiler [32] R package(v. 4.1.2) for detecting potentially upregulated pathways from KEGG database [33] and gene ontology terms. Similarly, significantly downregulated proteins (not less than 2-fold changes in control sample) plus unique control sample proteins were enriched in the same way to define potentially downregulated pathways and gene ontology terms.

## 3. Result and Discussion

### 3.1. Molecular Identification of MDR Human Pathogens

Phylogenetic analysis was conducted to confirm the taxonomic identity of the two multidrug-resistant isolates. The evolutionary history was inferred using the Neighbor-Joining method, and the robustness of the tree topology was assessed with bootstrap analysis based on 1000 replicates.

**Figure 1:**
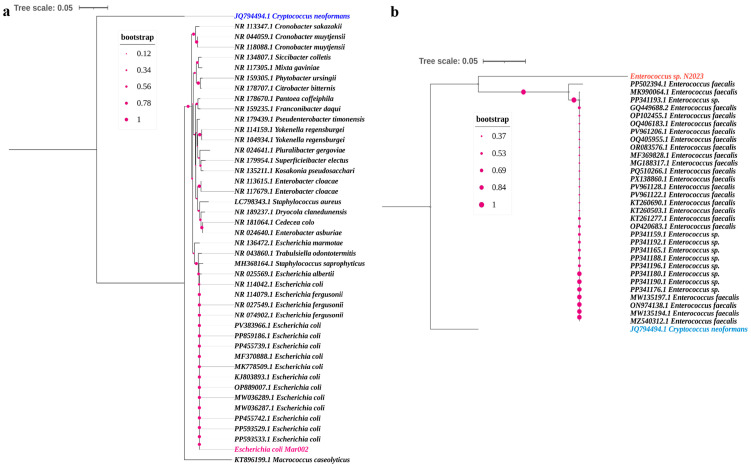
Phylogenetic analysis of the targeted isolates. Neighbor-Joining trees constructed from 16S rRNA gene sequences illustrate the phylogenetic position of (**a**) the *Escherichia coli* Mar002 isolate and (**b**) the *Enterococcus* sp. N2023 isolate among their respective genera. Reference sequences were obtained from the NCBI database. Bootstrap values (based on 1000 replicates) are shown at the branch points. The scale bar represents evolutionary distance.

The tree constructed for the first isolate (Figure 1a) clearly clusters it with reference sequences of *Escherichia coli*. The isolate *E. coli* Mar002 forms a distinct, well-supported clade with other *E. coli* strains, unequivocally confirming its species-level identification. The analysis was based on an alignment of 44 nucleotide sequences and 517 positions after the application of the complete deletion option.

Similarly, the phylogenetic tree for the second isolate (Figure 1b) places it within the genus *Enterococcus*. The isolate *Enterococcus* sp. N2023 groups robustly with sequences of Enterococcus faecalis, showing a close evolutionary relationship and high sequence similarity with this species. This analysis, encompassing 33 nucleotide sequences and 1167 positions, provides strong molecular evidence for its classification as an *Enterococcus* sp. isolate.

### 3.2. Characterization of Selenium Nanoparticles Produced by Ascorbic Acid and Citrate Ions

**Figure 2:**
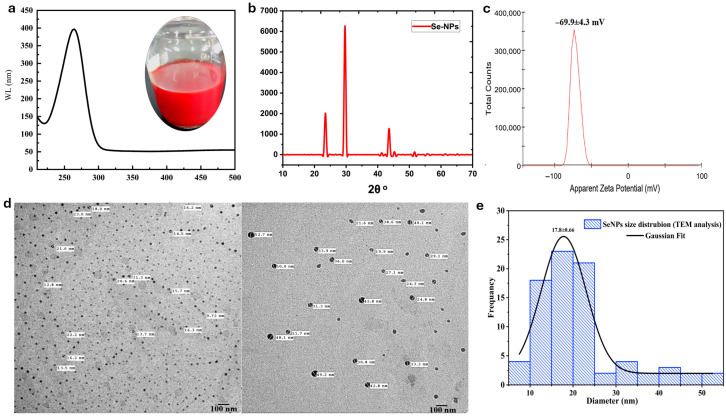
Morphological and physicochemical characterization of selenium nanoparticles (SeNPs). (**a**) UV-Vis spectroscopy; (**b**) Corresponding XRD patterns illustrate the crystallinity and phase of SeNPs; (**c**) Zeta potential of SeNPs. (**d**) Transmission Electron Microscopy (TEM) micrographs of SeNPs synthesized; (**e**) Distribution histogram of the size of SeNP-AA nanoparticles based on TEM images analyses.

The synthesis of selenium nanoparticles (SeNPs) was investigated using ascorbic acid (AA) and sodium citrate. Successful reduction of selenite (SeO_3_^2−^) to elemental selenium (Se^0^) was initially confirmed by a distinct red color and a characteristic surface plasmon resonance (SPR) peak at ~260 nm in reactions mediated by AA as a reducing agent (Figure 2a) [34].

The SeNP formulation was initially characterized using TEM, DLS, and XRD (Figure 2b–e). X-ray diffraction (XRD) analysis confirmed the synthesis of highly crystalline selenium nanoparticles (SeNPs) without any detectable impurities (Figure 2b) [35]. The diffraction pattern exhibited sharp and intense peaks at 2θ values of 23.45°, 29.65°, 41.27°, 43.58°, and 45.28°, corresponding to the (100), (101), (110), (102), and (111) lattice planes of pure trigonal (hexagonal) selenium. The average crystallite size, calculated from the peak broadening using the Debye–Scherrer equation, was approximately 10.97 nm, indicating the nanocrystalline nature of the particles. This pattern of high crystallinity and phase purity is consistent with previous studies showing that ascorbic acid is an effective reducing agent for crystalline SeNPs [17] and that citrate ions can serve as effective capping agents to promote uniform distribution and prevent aggregation [18]. For instance, a recent study by Alhawiti (2022) [18] on SeNPs synthesized using citric acid reported diffraction peaks at positions such as 2θ = 23.7 (100), 30.0 (101), 41.8 (110), 44.3 (102), 52.11 (112), 56.2 (202), and 61.7 (210). The sharp peak at 2θ = 30.0 (101) indicated a predominant orientation along the (101) facet and high purity, with a calculated crystallite size of approximately 37 nm as determined by the Debye–Scherrer equation [36]. The high zeta potential value of −69.9 ± 4.3 mV (Figure 2c) signifies a stable colloidal system, as values exceeding ±30 mV are generally indicative of electrostatic stabilization [37]. Comparatively, Shahabadi et al. (2021) [34] reported a zeta potential of −24.8 mV, a hydrodynamic size of 146 nm, and a polydispersity index (PDI) of 0.521 for SeNPs synthesized using ascorbic acid as both reducing and stabilizing agent. Transmission Electron Microscopy (TEM) analysis, which provides the core diameter of the primary particles in a dry state, revealed distinct morphologies for the SeNP formulation. SeNPs formed the smallest primary particles (17.8 ± 0.66 nm), which appeared well-dispersed on the TEM grid (Figure 2d,e). Literature reports consistently demonstrate that the size of selenium nanoparticles (SeNPs) is highly dependent on synthesis parameters. Shahabadi et al. [34] reported the formation of spherical SeNPs with a size of 134 nm using ascorbic acid as a reducing agent. Another comparative study demonstrated that using a 1:2 molar ratio of sodium selenite to ascorbic acid with bovine serum albumin (BSA) as a capping agent for 30 min consistently produced large SeNPs exceeding 700 nm, regardless of agitation. However, increasing the ascorbic acid ratio yielded significantly smaller, spherical nanoparticles in the 20–47 nm range, as confirmed by DLS and TEM [38]. Similarly, in biological synthesis, using *R. mucilaginosa* biomass over 48 h, the selenite oxyanion precursor concentration directly controlled the size of the resulting spherical SeNPs. SEM analysis showed that increasing the concentration from 2 mM to 5 mM led to a rise in average particle size from 103 to 118 nm to 363 nm [39]. These physicochemical properties of the synthesized NPs suggest that the synthesized nanoparticles exhibit a stable and robust capping layer, where ascorbic acid contributes strong reducing power, while citrate ions serve to stabilize the nanoparticle surface through electrostatic repulsion, producing discrete and stable nanoparticles.

### 3.3. Antimicrobial Activity, MIC, MBC, and Bactericidal Activity of the Fabricated Nano Selenium Particles Against Pathogenic Bacteria

**Figure 3:**
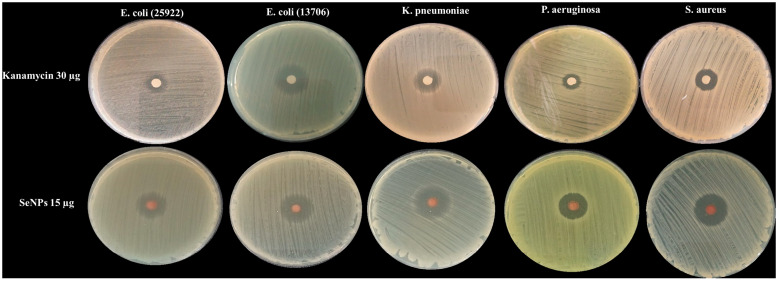
Representative images of agar plates showing the inhibition zones against human pathogenic bacteria after being treated with SeNPs and Kanamycin.

The strain-specific antibacterial activity of selenium nanoparticles (SeNPs) synthesized with ascorbic acid and citrate ions was investigated. The results, detailed in Figure 3 and Table 1, demonstrated a significant variation in susceptibility among the tested bacterial pathogens.

SeNPs exhibited the most potent efficacy against *Staphylococcus aureus* and *Klebsiella pneumoniae*, generating the largest inhibition zones of 22.33 ± 0.58 mm and 22.00 ± 1.00 mm, respectively. In contrast, the lowest antibacterial activity among the standard strains was observed against *Escherichia coli* (25922) and *Pseudomonas aeruginosa*, with inhibition zones of 18.33 ± 1.53 mm and 18.67 ± 0.58 mm. A notably pronounced resistance was displayed by multidrug-resistant (MDR) bacteria. *Enterococcus* sp. showed a markedly small inhibition zone of 7.3 ± 0.58 mm, while MDR *E. coli* Mar demonstrated an intermediate susceptibility with a zone of 17.7 ± 1.15 mm.

Further quantification of antimicrobial potency through Minimum Inhibitory Concentration (MIC) and Minimum Bactericidal Concentration (MBC) assays corroborated the strain-dependent activity (Table 1). SeNPs were most effective against *S. aureus*, *E. coli* (13706), and *K. pneumoniae*, with a consistent MIC of 4.686 µg/mL. The MBC values for these strains were 18.744 µg/mL for *S. aureus* and *E. coli* (13706) and 9.372 µg/mL for *K. pneumoniae*, resulting in MBC/MIC ratios of 2 to 4, indicating a bactericidal effect. In comparison, a higher MIC of 9.374 µg/mL was required for *E. coli* (25922) and the *MDR E. coli* Mar, with a corresponding MBC of 37.496 µg/mL (MBC/MIC ratio = 4). *P. aeruginosa* (27853) exhibited even lower susceptibility, with a MIC of 18.75 µg/mL and an MBC of 37.496 µg/mL. Most significantly, the MDR *Enterococcus* sp. was highly resistant, requiring a substantially higher MIC of 75 µg/mL and an MBC of 300 µg/mL to achieve inhibition and killing, respectively.

In terms of the positive control, kanamycin, all pathogens showed intermediate susceptibility, except for *E. coli* (13706), which was sensitive, and UTI-isolated pathogens, *E. coli* (25922) and *P. aeruginosa*, were resistant, in line with the CLSI guidelines.

The significant bactericidal efficacy of selenium nanoparticles (SeNPs) against a diverse spectrum of bacterial pathogens, as demonstrated in this study, underscores their potential as promising antimicrobial agents. This aligns with a growing body of literature, which indicates that the antimicrobial properties of SeNPs are highly contingent on factors such as nanoparticle concentration, the specific microbial species, and the synthesis method [40]. For instance, Cremonini et al. [41] reported that SeNPs synthesized by *Stenotrophomonas maltophilia* and *Bacillus mycoides* inhibited *P. aeruginosa* at concentrations ranging from 8 to 512 mg/mL. Similarly, El-Deeb et al. [42] found that SeNPs synthesized by *Providencia vermicola* were ineffective against certain Gram-negative pathogens but showed strong antibacterial activity against *S. aureus* and *Bacillus cereus*. Furthermore, research by Greeshma and Mahesh [43] and Alam et al. [44] confirms that activity against pathogens like *E. coli*, *S. aureus*, and *P. aeruginosa* is concentration-dependent, with efficacy observed across a wide range (from 1 to 10 µg/mL to 400 µg/mL). However, this activity is not universal; studies by Cremonini et al. [41] and El-Deeb et al. [42] report species-specific effects, where SeNPs inhibited *P. aeruginosa* and *S. aureus* but were ineffective against certain *Candida* species and other Gram-negative bacteria. A critical determinant of this variable susceptibility is the surface chemistry of the nanoparticles. As noted by Escobar-Ramírez et al. [40], the size, charge, and surface coating of SeNPs are pivotal. The observed potent antibacterial activity of our SeNPs, despite their high negative zeta potential conferred by a citrate shell, highlights the complex interplay between surface charge and biological activity. While conventional wisdom, supported by Galić et al. [45] and Rangrazi et al. [46], suggests that a positive zeta potential enhances interaction with negatively charged bacterial membranes, our experimental data reveals a significant advantage of our specific SeNP formulation. Contrary to the expectation that cationic nanoparticles are more cytotoxic on mammalian cells than those with neutral or negative surface charge [47], our findings are consistent with research indicating that negatively charged nanoparticles can exhibit potent antimicrobial activity with reduced toxicity, a crucial feature for pharmaceutical applications [48]. In their study, Salvioni et al. [48] demonstrated that negatively charged SeNPs (BSA-coated and undefined coating) could severely impair bacterial cell growth and viability in both Gram-negative (*Stenotrophomonas bentonitica*) and Gram-positive (*Lysinibacillus sphaericus*) models. Notably, the undefined coating SeNPs displayed the highest percentage of dead cells (85−91%) and caused nearly 80% DNA degradation, effects that were linked to a significant increase in reactive oxygen species (ROS) production. Collectively, this evidence positions our citrate-capped SeNPs as part of an effective class of antimicrobials whose potent, broad-spectrum action is uniquely enabled by their anionic surface chemistry, offering a promising safety advantage for potential applications.

### 3.4. Transmission Electron Microscope for Estimating Antimicrobial Activity of SeNPs

**Figure 4:**
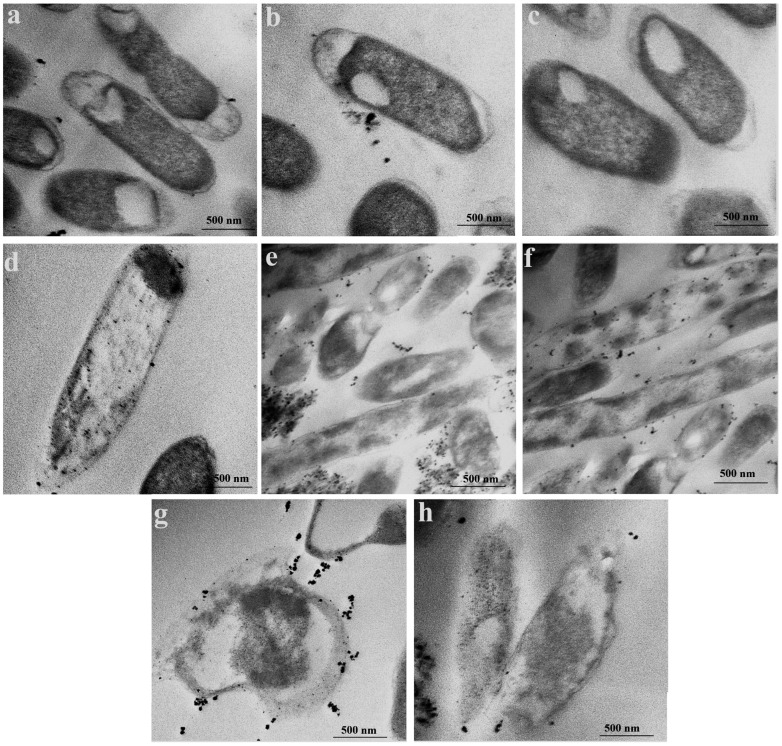
Transmission electron microscopy images of MDR E. coli cells treated with selenium nanoparticles (SeNPs). (**a**–**c**) control (untreated) cells; (**d**–**h**) SeNP-treated *E. coli* cells.

Transmission electron micrograph analysis reveals the cytotoxic effect of selenium nanoparticles (SeNPs) on MDR *E. coli* cells, as illustrated in Figure 4. Untreated control cells (Figure 4a–c) display intact, rod-shaped morphology with well-defined membranes and undisturbed internal architecture. In contrast, SeNP exposure induces progressive and severe cellular degradation (Figure 4d–h). The accumulation of SeNPs on the bacterial cell envelope (Figure 4d–f), despite their mutually negative surface charges, suggests that stronger, short-range forces may mediate the initial attachment. This observed adhesion can be interpreted within the framework of colloidal interaction theory (DLVO theory), where attractive forces such as van der Waals interactions and hydrophobic effects may dominate at close range, potentially facilitating adhesion despite overall electrostatic repulsion [49]. This surface association compromises membrane integrity by altering permeability, disrupting normal transport processes, and impairing membrane-bound enzyme activity [50]. Also, the small size of the SeNPs enables them to traverse the bacterial cell envelope [51]. In *E. coli*, which possesses a thin peptidoglycan layer and an outer membrane, SeNPs are hypothesized to infiltrate either by passive diffusion through the lipid bilayer or via pore penetration [52]. Once internalized, SeNPs instigate substantial intracellular disruption, directly contributing to a loss of cell viability [51].

Advanced stages of damage are evident in Figure 4g,h, which show rupture of the cell wall and membrane. This breach of the bacterial envelope facilitates the extensive infiltration of SeNPs into the cytoplasm, where they form dense aggregates. This physical disruption is consistent with literature findings that nanoparticles can induce pore formation and membrane lysis, leading to the leakage of cytoplasmic contents and catastrophic failure of essential cellular functions [53].

The primary mechanism underpinning this observed damage is likely oxidative stress. Intracellular SeNPs can generate reactive oxygen species (ROS), which cause peroxidation of lipids, denaturation of proteins, and damage to DNA, ultimately triggering cell death [54]. This oxidative assault is a plausible explanation for the initial membrane compromise and the subsequent cytoplasmic disintegration.

An additional contributory mechanism involves the release of toxic ions. In an aqueous environment, SeNPs may dissolve, releasing hydrated selenium ions. These ions can exert antimicrobial effects by inactivating critical intracellular enzymes, interfering with bacterial signal transduction pathways—particularly through disruption of phosphorylation—and obstructing nucleic acid metabolism by binding to DNA phosphorus residues, thereby hindering replication and transcription [55,56].

### 3.5. Proteomic Analysis

**Figure 5:**
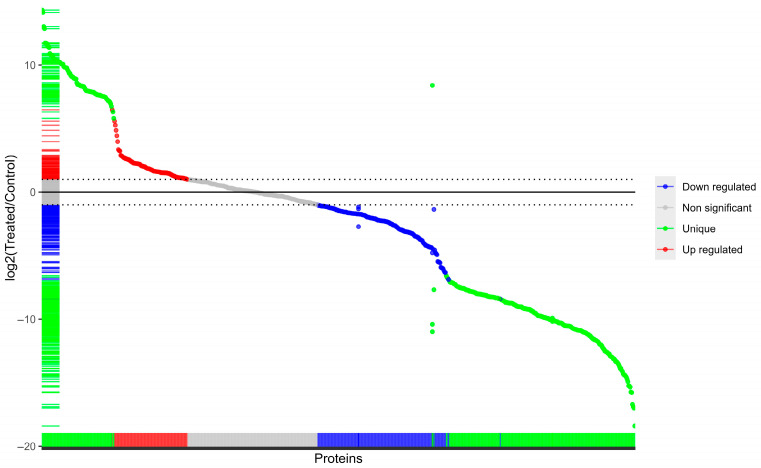
A volcano plot depicting the differential expression of proteins in SeNPs-treated MDR *E. coli* cells compared to control cells.

Proteomic profiling data provides a comprehensive view of how SeNPs remodel the cell’s protein expression. The treatment induced a pronounced shift, with a total of 268 proteins undergoing significant changes. Notably, the number of downregulated proteins (172) was nearly double that of upregulated ones (96), hinting at a potential widespread suppression of certain cellular processes or pathways (Figure 5). The proteomic analysis identified proteins that were exclusively present in one condition and completely absent in the other. Specifically, 247 proteins were uniquely detected in the control cells, while 94 proteins were uniquely expressed in the SeNPs-treated cells.

This condition-specific expression indicates a fundamental reprogramming of the cellular proteome, where SeNPs treatment not only modulates the levels of existing proteins but also leads to the suppression of one set of pathways and the de novo activation of another.

### 3.6. GO Analysis from Proteomic Analysis

**Figure 6:**
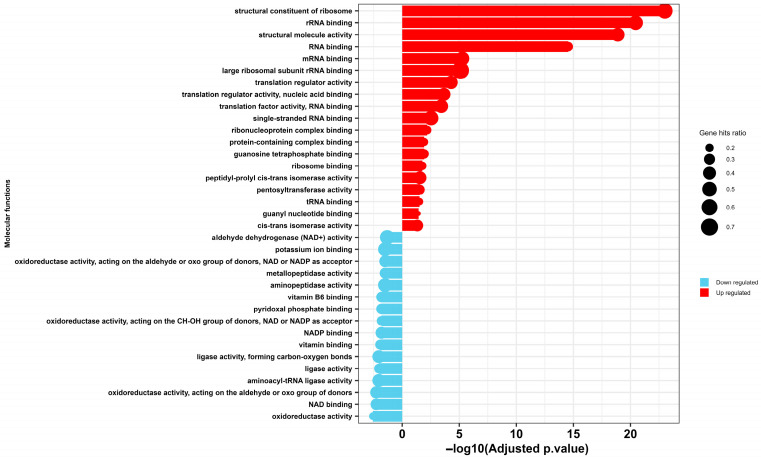
Gene Ontology (GO) enrichment analysis of molecular functions in MDR *E. coli* following treatment with selenium nanoparticles (Se NPs). The chart depicts significantly over-represented GO terms among differentially expressed proteins, categorized as either upregulated (red) or downregulated (blue).

GO enrichment analysis of molecular function reveals that selenium nanoparticle (SeNP) exposure induces a profound and systematic dysregulation of essential molecular functions in *E. coli*, culminating in catastrophic cellular failure (Figure 6). The observed profile delineates a clear mechanistic pathway of toxicity, characterized by a futile compensatory stress response operating in parallel with a critical collapse of core metabolic and biosynthetic processes.

The data indicates a significant upregulation of genes encoding the structural constituents of the ribosome, as well as factors involved in rRNA, mRNA, and tRNA binding and regulation. This is interpreted not as a sign of proliferative health, but rather as an integrated cellular stress response, potentially mediated by the alarmone (p)ppGpp [57], to widespread nanoselenium-induced damage. This model is supported by findings in *Pseudomonas aeruginosa*, where similar upregulation of transcriptional and translational machinery under nanoparticle stress represents a desperate attempt to maintain homeostasis [58]. The concurrent upregulation of peptidyl-prolyl cis-trans isomerase activity may indicate a crisis in protein folding [59]. In essence, the cell mounts a heroic effort to replace a damaged proteome by hyper-activating its protein synthesis apparatus.

However, this compensatory effort is rendered futile by the simultaneous and catastrophic downregulation of fundamental metabolic pathways. A coordinated suppression of oxidoreductase activities—specifically those acting on aldehyde/oxo and CH-OH groups with NAD(P)+ cofactors, including aldehyde dehydrogenase—reveals a multi-pronged attack on cellular viability. This suppression cripples central energy-yielding pathways such as glycolysis, leading to severe ATP depletion [60]. Furthermore, it impairs the detoxification of reactive aldehydes and, crucially, disrupts the regeneration of NADPH [60]. The latter effect catastrophically compromises the bacterial antioxidant system, as NADPH is essential for replenishing reduced glutathione [61], leaving the cell defenseless against SeNP-induced oxidative stress.

The toxic insult extends further to cripple biosynthetic capacity and ion homeostasis. The downregulation of potassium ion binding proteins suggests a disruption of membrane potential and cellular turgor, fundamental to the cell’s bioenergetic state [62]. Simultaneously, the suppression of vitamin B6 [63] and pyridoxal phosphate binding proteins [64] strikes a critical blow to amino acid metabolism and one-carbon units, halting the production of vital biomolecules. Most decisively, the collective downregulation of aminoacyl-tRNA ligases and aminopeptidase activity represents a targeted shutdown of the protein life cycle [65]. This disruption prevents the charging of tRNAs, halting translation initiation, while also impairing protein maturation and the recycling of amino acids.

**Figure 7:**
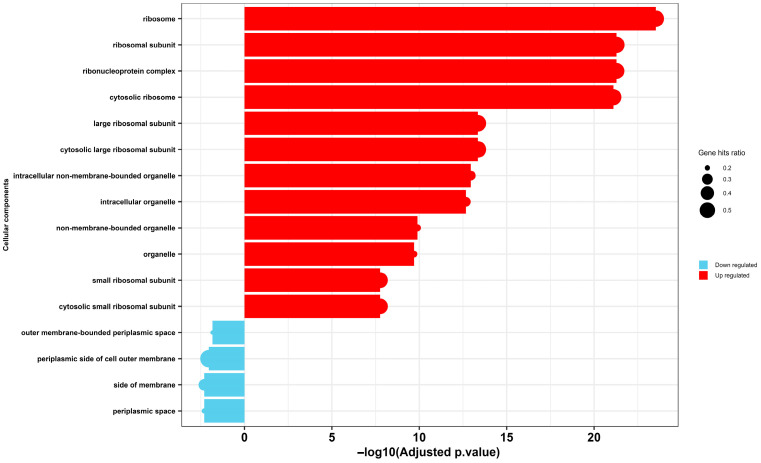
Gene Ontology (GO) enrichment analysis of cellular components of MDR *E. coli* following treatment with selenium nanoparticles (Se NPs). The chart depicts significantly over-represented GO terms among differentially expressed proteins, categorized as either upregulated (red) or downregulated (blue).

Similarly, Gene Ontology (GO) enrichment analysis of cellular components revealed a stark dichotomy in the transcriptional response of *Escherichia coli* to selenium nanoparticle (SeNP) exposure (Figure 7). A significant upregulation was observed for terms associated with the protein synthesis machinery, including the ribosome, ribosomal subunits, and ribonucleoprotein complexes. This tooling up of the translational apparatus is a recognized hallmark of the bacterial stress response, as the cell reallocates resources to produce a defensive arsenal of chaperones, detoxification enzymes, and repair proteins to mitigate damage [66]. Conversely, a significant downregulation was detected for components of the cell envelope, specifically the periplasmic space and the outer membrane-bounded periplasmic space. This transcriptional signature finds direct correlation in our transmission electron microscopy (TEM) analysis (Figure 4), which visually confirmed the accumulation of Se NPs within the periplasmic space and their subsequent aggregation inside the cells, coinciding with severe internal disruption and membrane rupture. The downregulation of envelope biogenesis genes, therefore, is likely a direct consequence of a failed compensatory response; the physical damage inflicted by the SeNPs—as witnessed by the membrane rupture—overwhelms the cell’s capacity to maintain and repair its outer structures, leading to a collapse of transcriptional programs for these components.

The Gene Ontology enrichment analysis reveals a coherent and pronounced biological response in *Escherichia coli* following treatment with selenium nanoparticles (SeNPs) (Table 2, Supplementary Figure S1). The transcriptional profile is dominated by a pervasive downregulation of core metabolic pathways, indicating a severe disruption of the cell’s energy and biosynthetic capabilities. This includes the collective suppression of Energy Metabolism and Cellular Respiration (184 genes), Carboxylic and Organic Acid Metabolism (120 genes), Nucleotide and Cofactor Metabolic Processes (49 genes), and Amino Acid Metabolic Processes (78 genes for biosynthesis), painting a clear picture of a global metabolic shutdown. This pattern is consistent with the established mechanism of metal and metalloid nanoparticles, such as silver nanoparticles (AgNPs), which are known to induce oxidative stress, disrupt the proton motive force, and collapse ATP synthesis, leading to the downregulation of energy-dependent processes [67]. The parallel downregulation of Translation and Protein Synthesis Machinery (33 genes) and Cellular Assembly and Biogenesis (72 genes) further confirms a halt in growth and proliferation, representing a classic stringent response to conserve resources under duress [68].

Conversely, the most prominent upregulated responses suggest a concerted, compensatory effort to cope with the induced damage. A massive investment in the protein synthesis apparatus is evident from the strong upregulation of Translation and Protein Synthesis Machinery (70 genes), Ribosome Biogenesis (45 genes), and Cellular Assembly and Biogenesis (115 genes), likely to replace proteins damaged by SeNP-induced oxidative stress. This is accompanied by a metabolic rewiring towards alternative energy sources, seen in the upregulation of Glycerol and Polyol Metabolism (7 genes) and specific Amino Acid Catabolic processes (12 genes), a strategy bacteria use to bypass disrupted central pathways like the TCA cycle [69]. The significant upregulation of the Cellular Stress Response (27 genes, specifically to heat/temperature) and Post-transcriptional Regulation (18 genes) points to damage to the cell envelope and a need to fine-tune the costly process of translation, consistent with physical membrane damage caused by various nanoparticles [8,70]. Furthermore, the upregulation of Nucleotide Metabolism (18 genes) provides the essential RNA building blocks required to support the observed surge in ribosome production.

### 3.7. KEGG Analysis from Proteomic Analysis

To further elucidate the metabolic consequences of selenium nanoparticle (SeNP) stress on *E. coli*, we performed KEGG pathway enrichment analysis on the downregulated proteome. The results, detailed in Table 3, provide a granular view of the specific metabolic pathways most severely impacted, strongly supporting the GO term analysis and confirming a state of metabolic arrest.

The most striking finding was the comprehensive suppression of central carbon metabolism. A total of 42 proteins involved in the broad category of Carbon metabolism were significantly downregulated (adjusted *p*-value = 2.50 × 10^−9^). This was reflected in the specific inhibition of all major glycolytic and energy-yielding pathways: Glycolysis/Gluconeogenesis (21 proteins), the Citrate cycle (TCA cycle) (16 proteins), and Pyruvate metabolism (22 proteins). The concurrent downregulation of the Pentose phosphate pathway and Oxidative phosphorylation (13 proteins) indicates a collapse of both energy (ATP) and reducing power (NADPH) production. This coordinated shutdown suggests that Se NPs directly or indirectly target the core engine of the bacterial cell, crippling its ability to generate the fundamental precursors and energy required for growth and proliferation [67].

Metabolic paralysis extends beyond energy production to the biosynthetic processes that depend on it. Pathways for the Biosynthesis of amino acids (26 proteins) and the Biosynthesis of cofactors (23 proteins) were significantly enriched among downregulated proteins. This includes specific pathways for Arginine and proline metabolism and Cysteine and methionine metabolism, which are crucial for maintaining cellular redox balance. The downregulation of Aminoacyl-tRNA biosynthesis, although not statistically significant after adjustment, aligns with the GO analysis showing a suppression of translation machinery. This collective impairment of anabolic pathways demonstrates that the cell is not only unable to produce energy but is also blocked from synthesizing the essential building blocks for proteins, nucleic acids, and enzymatic cofactors, leading to a comprehensive stasis.

The impact of SeNP stress is not confined to central metabolism but permeates broader cellular functions. The significant downregulation of pathways for the Biosynthesis of secondary metabolites (82 proteins) and Microbial metabolism in diverse environments (69 proteins) underscores a global failure to adapt and sustain complex metabolic networks. Furthermore, the downregulation of Glutathione metabolism (10 proteins, Table 4) is particularly noteworthy. Glutathione is a key antioxidant, and its impaired metabolism suggests the cell’s primary defense against oxidative stress is compromised, potentially exacerbating the damage caused by SeNP-induced reactive oxygen species (ROS) [68]. Interestingly, pathways for membrane transport (ABC transporters) and signal transduction (Two-component systems) were not significantly enriched, suggesting that the primary mode of toxicity is not a simple disruption of nutrient uptake but a direct assault on intracellular metabolic integrity.

Finally, the quantitative proteomic analysis provides definitive evidence that selenium nanoparticle (SeNP) exposure triggers a catastrophic failure of the oxidative stress response in *E. coli* by specifically targeting the glutathione system (Table 4) and key metabolic and redox pathways (Table 5). Selenium nanoparticles (SeNP) exposure induces a concerted and catastrophic failure in *E. coli* by simultaneously dismantling both the cellular antioxidant defense system and core metabolic pathways, creating a lethal feedback loop. The primary defensive barrier, the glutathione system, is specifically targeted, with its key components—glutathione S-transferases (GstA/GstB) and glutaredoxin 2 (GrxB)—depleted by 18 to 19-fold, and the stress-related protein HchA is dramatically reduced by over 63-fold. This effectively disarms the cell’s capacity to neutralize peroxides and repair oxidative damage. Concurrently, central metabolic pathways suffer severe disruption; iron–sulfur cluster-dependent enzymes in the Citrate Cycle, such as Aconitate hydratase (AcnB) and Succinate dehydrogenase (SdhA), are downregulated by 8.1 to 13.9-fold, while the Oxidative Phosphorylation pathway is impaired, with a 4.7-fold decrease in NADH dehydrogenase subunit B (NuoB). The resulting malfunction of the electron transport chain likely exacerbates reactive oxygen species (ROS) generation. Consequently, the cell is trapped in a vicious cycle: the compromised metabolism produces more ROS, while the crippled glutathione system is incapable of mounting an effective defense. This self-amplifying cycle of escalating oxidative stress and irreversible metabolic damage ultimately leads to complete cellular collapse and death. It is important to note that the hypothesized antimicrobial mechanism of SeNPs against MDR *E. coli*, as derived from this proteomic screen, requires direct validation through focused follow-up experiments, including genetic and biochemical approaches.

## 4. Conclusions

This study establishes a robust, green synthesis protocol for highly stable and crystalline selenium nanoparticles (SeNPs) with potent, broad-spectrum antibacterial activity against multidrug-resistant pathogens. Building on this foundation, we propose a detailed and testable mechanistic hypothesis to explain their efficacy. We posit that SeNP activity is not a singular event but a cascade initiated by non-electrostatic attachment and membrane compromise, leading to a catastrophic intracellular cycle. Our integrative analysis, combining phenotypic assays with preliminary proteomic profiling, suggests this cycle is driven by a synergistic dual assault: SeNPs appear to simultaneously induce overwhelming oxidative stress while strategically dismantling the cell’s primary glutathione-based antioxidant defense and crippling central energy metabolism. The resulting lethal feedback loop—where impaired respiration exacerbates ROS production and a disabled repair system fails to mitigate damage—provides a coherent model for the observed rapid metabolic paralysis and cell death. We present this as a central hypothesis generated from our data. While the compelling potency of the SeNPs is firmly established, we note that the specific proteomic model, derived from an initial single-replicate analysis, requires future validation with replicated omics and genetic experiments. Nonetheless, this work moves beyond a simple efficacy report by providing a definitive molecular framework that explains the antibacterial action of SeNPs and positions them as a sophisticated, multi-targeted therapeutic strategy against the mounting antimicrobial resistance crisis.

### Limitation

The proteomic study, designed as an initial discovery screen, lacked biological replication. Consequently, while the substantial alterations in core pathways offer compelling, hypothesis-generating evidence for the SeNPs mechanism, the findings for individual proteins with lower fold changes require validation through targeted follow-up experiments such as genetic or biochemical assays. Furthermore, the nanoparticles were characterized in their purified state, but their stability within the biological assay medium was not directly assessed. The reproducible dose–response observed across antimicrobial, morphological, and proteomic assays implies the NPs remained active, yet future studies incorporating in situ stability measures should be considered to fully define the operative nano-form.

## Figures and Tables

**Table 1:** Inhibition Zone (IZ), MIC, MBC, and MBC/MIC ratios of SeNPs and Kanamycin against human pathogenic bacteria. The data are the mean of three replicates. The statistical significance of differences in Inhibition Zone (IZ) measurements was determined using a two-way ANOVA test in GraphPad Prism 9 software.

	SeNPs				Kanamycin			
	IZ (mm)	MIC (µg/mL)	MBC (µg/mL)	MBC/MIC	IZ (mm)	MIC (µg/mL)	MBC (µg/mL)	MBC/MIC
*Enterococcus* sp.	7.300 ± 0.58	75	300	4	11.00 ± 1.00	1875	3750	2
*E. coli Mar002*	17.70 ± 1.15	9.374	37.496	4	8.000 ± 1.00	468.75	937.5	2
*E. coli* (25922)	18.33 ± 1.53	9.374	37.496	4	13.00 ± 1.00	468.75	1875	4
*S. aureus* (12600)	22.33 ± 0.58	4.686	18.744	4	17.30 ± 0.58	14.65	58.6	4
*P. aregonosa* (27853)	18.67 ± 0.58	18.75	37.496	4	13.00 ± 0.58	58.6	117.2	2
*E. coli* (13706)	19.00 ± 1.00	4.686	18.744	4	18.00 ± 1.00	3.66	7.32	2
*K. penoumonia* (43816)	22.00 ± 1.00	4.686	9.372	2	17.00 ± 1.00	14.65	29.3	2
Two-way ANOVA (*p*-value)	<0.0001				<0.0001			

**Table 2:** Summary of Significantly Enriched Downregulated and Upregulated Biological Processes from GO Enrichment Analysis of MDR *E. coli* Treated with Selenium Nanoparticles. The gene ratio hits and the -log 10 (adjusted *p*-value for each process) are provided in Figure S1.

Biological Process	Total Downregulated Genes	Total Upregulated Genes
Energy Metabolism and Cellular Respiration *(TCA cycle, Oxidative phosphorylation, Aerobic respiration, Generation of precursor metabolites and energy)*	184	–
Catabolic Processes (*Cellular catabolism, Organic/carboxylic acid catabolism, Small molecule catabolism)*	95	–
Carboxylic and Organic Acid Metabolism	120	–
Amino Acid Metabolic Processes	78 (Amino acid metabolic process, Biosynthesis)	12 (Amino acid catabolic process)
Nucleotide and Cofactor Metabolic Processes	49 (Nicotinamide/Pyridine nucleotide metabolic process)	18 (Nucleoside monophosphate metabolic process)
Translation and Protein Synthesis Machinery *(Translation, Translational initiation, tRNA aminoacylation, tRNA metabolic process)*	33 (tRNA aminoacylation, Translation, tRNA metabolic process)	70 (cytoplasmic translation, translation, translational initiation, tRNA aminoacylation)
Cellular Assembly and Biogenesis *(Protein complex/protein–RNA complex assembly, Ribosome assembly, Organelle assembly, Cellular component biogenesis)*	72 (Protein-containing complex assembly, Protein complex oligomerization, Ribosome assembly)	115 (ribonucleoprotein complex biogenesis, organelle assembly, cellular component biogenesis)
Ribosome Biogenesis *(Ribosome biogenesis, Ribosomal subunit assembly, Ribonucleoprotein complex biogenesis)*	–	45
Cellular Stress Response	45 (Response to oxidative stress)	27 (Response to heat and temperature stimulus)
Post-transcriptional Regulation *(Regulation of translation, post-transcriptional gene expression)*	–	18
Glycerol and Polyol Metabolism	–	7

**Table 3:** KEGG pathways significantly enriched for downregulated proteins in MDR *E. coli* under selenium nanoparticle (SeNPs) stress.

Category	Pathway Name	Downregulated Proteins	Total	Adjusted *p*-Value
Central Carbon Metabolism	Carbon metabolism	42	171	2.50 × 10^−9^
	Glycolysis/Gluconeogenesis	21		1.97 × 10^−6^
	Pyruvate metabolism	22		3.62 × 10^−5^
	Citrate cycle (TCA cycle)	16		1.97 × 10^−6^
	Pentose phosphate pathway	10		0.0279
	Glyoxylate and dicarboxylate metabolism	13		0.0141
	2-Oxocarboxylic acid metabolism	13		0.00108
	Amino sugar and nucleotide sugar metabolism	14		0.00814
	Biosynthesis of nucleotide sugars	10		0.0542
	Starch and sucrose metabolism	10		0.141
Amino Acid Metabolism	Biosynthesis of amino acids	26	57	0.0227
	Arginine and proline metabolism	11		0.00141
	Cysteine and methionine metabolism	10		0.0632
	Aminoacyl-tRNA biosynthesis	10		0.986
Cofactor Biosynthesis	Biosynthesis of cofactors	23	23	0.447
Energy Metabolism	Oxidative phosphorylation	13	40	0.0141
	Other carbon fixation pathways	15		0.000187
	Methane metabolism	12		0.00499
Membrane Transport System	ABC transporters	20	20	0.984
Signal Transduction	Two-component system	10	10	0.96
Virulence and Oxidative Stress Pathways	Glutathione metabolism	10	17	0.00438
	beta-Lactam resistance	3		0.679
	Cationic antimicrobial peptide (CAMP) resistance	4		0.953
Broad Metabolic Networks	Biosynthesis of secondary metabolites	82	177	8.04 × 10^−7^
	Microbial metabolism in diverse environments	69		9.81 × 10^−7^
	Propanoate metabolism	14		0.000549
	Butanoate metabolism	12		0.00783

**Table 4:** Quantitative Profiling of Significantly Downregulated Glutathione Metabolism Proteins in *E. coli* under Selenium Nanoparticle Stress.

Gene ID	Protein Name	Log2 FC	Fold Decrease (1/FC)	*p*.Adjust
gstB	Glutathione S-transferase B	−4.2	18.4	0.00438
gloA	Lactoylglutathione lyase	−0.17	1.1	
grxA	Glutaredoxin 1	−0.39	1.3	
hchA	Protein hchA	−5.98	63.3	
gstA	Glutathione S-transferase GstA	−4.2	18.4	
ggT	Gamma-glutamyltranspeptidase	−3.51	11.4	
grxB	Glutaredoxin 2	−4.25	19.1	
grxC	Glutaredoxin 3	−3.26	9.6	

**Table 5:** Proteomic Profiling of Key Metabolic and Redox Pathways in *E. coli* Under Selenium Nanoparticle Stress.

	Role in Oxidative Stress	*p*.Adjust	Protein Name	Log2FC	Fold Decrease
Oxidative Phosphorylation	Primary ROS Generator	0.014	nuoB (NADH/ubiquinone oxidoreductase subunit B)	−2.244	4.7
			atpD (F0F1 ATP synthase subunit beta)	0.045	0.97
			sdhA (Succinate dehydrogenase flavoprotein subunit)	−3.8	13.9
			sdhB (Succinate dehydrogenase iron–sulfur subunit)	−2.62	6.1
Citrate Cycle/TCA Cycle	ROS Generator and Sensitive Target	1.97 × 10^−6^	acnB (Aconitate hydratase 2)	−3.016	8.1
			sdhA (Succinate dehydrogenase flavoprotein subunit)	−3.8	13.9
			icd (Isocitrate dehydrogenase)	−2.729	6.6
			mdh (Malate dehydrogenase)	−1.507	2.8
Glycolysis/Gluconeogenesis	Sensitive Target	1.97 × 10^−6^	gapA (Glyceraldehyde-3-phosphate dehydrogenase A)	−0.032	1.02
			tpiA (Triosephosphate isomerase)	−0.321	1.25
			eno (Enolase)	−0.154	1.11
			pykF (Pyruvate kinase I)	−1.147	2.2
Biosynthesis of Cofactors	Essential for Antioxidant Defenses	0.447	gstA (Glutathione S-transferase A)	−4.201	18.5
			ggt (Gamma-glutamyltranspeptidase)	−3.507	11.4
			iscS (Cysteine desulfurase)	−2.052	4.1
			panC (Pantothenate synthetase)	−1.115	2.2

## Data Availability

The original contributions presented in this study are included in the article/Supplementary Materials. Further inquiries can be directed to the corresponding authors.

## Supplementary Materials

The following supporting information can be downloaded at https://www.mdpi.com/article/10.3390/microorganisms14010089/s1, Figure S1: The gene ratio hits and the −log 10 (adjusted *p*-value) of Significantly Enriched Downregulated and Upregulated Biological Processes from GO Enrichment Analysis of MDR *E. coli* Treated with Selenium Nanoparticles.

## Abbreviations

The following abbreviations are used in this manuscript:

SeNPs	Selenium nanoparticles
ROS	Reactive Oxygen Species
TEM	Transmission Electron Microscope
AMR	Antimicrobial resistance
