# Supplementary figures and images for: Bactericidal Activity of Selenium Nanoparticles Against a Multidrug-Resistant Pathogen: Mechanistic Hypothesis from Exploratory Proteomics
Source: Microorganisms. 2025 Dec 31;14(1):89. doi: 10.3390/microorganisms14010089 (PMC12844374; doi:10.3390/microorganisms14010089)

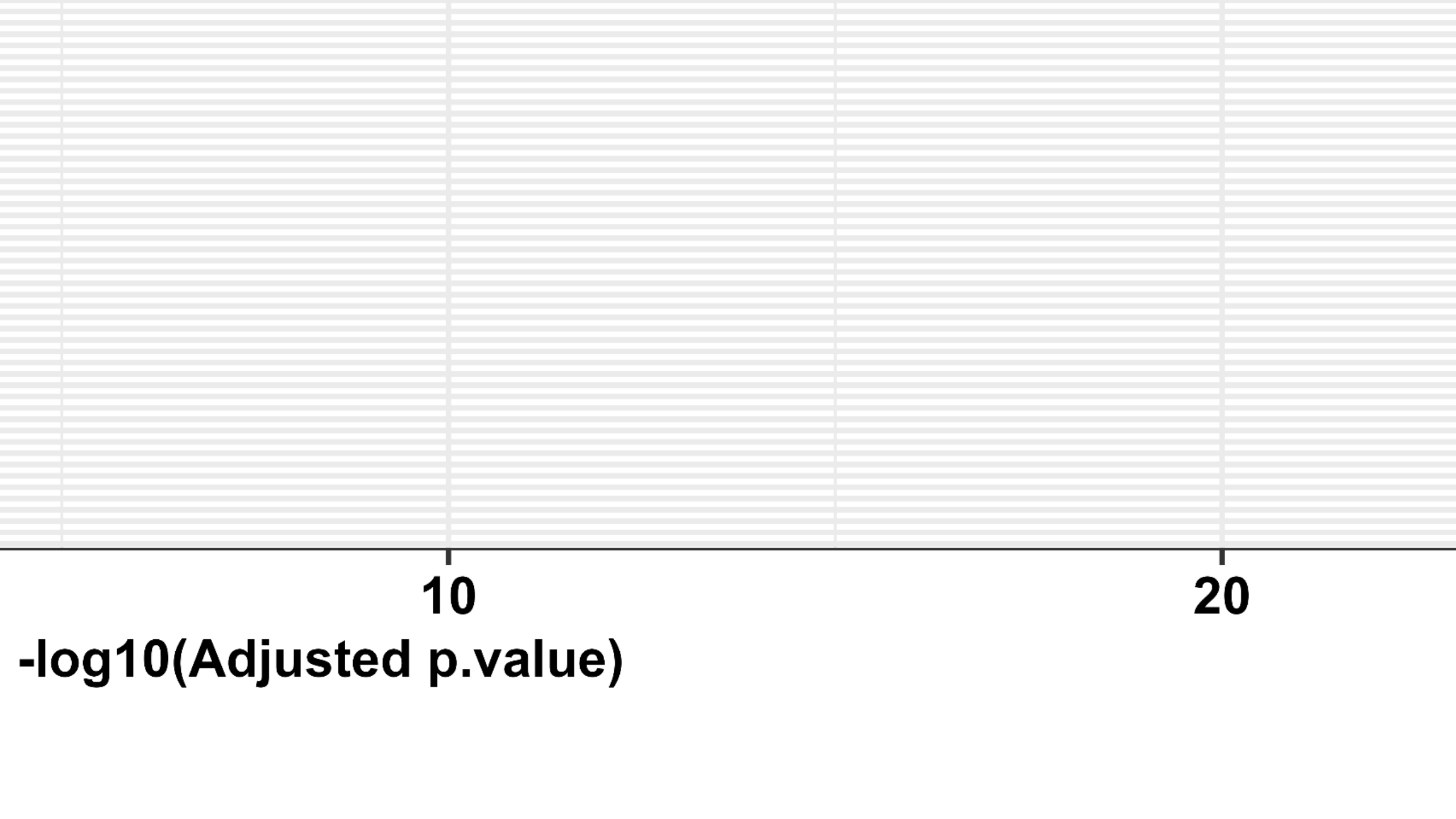

Supplement: Supplementary file 1 [file microorganisms-14-00089-s001.zip › microorganisms-4053624-supplementary.pdf]
